# Jejunal Gastrointestinal Stromal Tumor: A Diagnostic Challenge

**DOI:** 10.7759/cureus.38098

**Published:** 2023-04-25

**Authors:** Edison D Miranda, Tatiana Fernandez Trokhimtchouk, Luis F Flores, Álvaro Morillo Cox, Jose R Negrete

**Affiliations:** 1 Internal Medicine, Universidad Internacional del Ecuador, Axxis Hospital, Quito, ECU; 2 General Surgery, Universidad Internacional del Ecuador, Axxis Hospital, Quito, ECU

**Keywords:** multidisciplinary approach, laparoscopy, endoscopy, gastrointestinal bleeding, gist

## Abstract

Gastrointestinal stromal tumors (GISTs) are a common type of soft tissue sarcoma that originates from the interstitial cells of Cajal in the gastrointestinal (GI) tract. These tumors usually affect people above 50 years of age and can be difficult to diagnose, as symptoms can be vague and nonspecific, with some patients remaining asymptomatic. Early diagnosis and treatment are crucial because GISTs can be aggressive and may metastasize.

We present a case of a 74-year-old man who presented to our hospital with GI bleeding and anemia. Despite initial investigations, the source of bleeding was not identified until capsule endoscopy and then balloon enteroscopy revealed an ulcerated mass in the jejunum. The tumor was successfully removed using a minimally invasive laparoscopic approach, and the histopathologic report confirmed the diagnosis of GIST. The patient had an uneventful postoperative course.

This case highlights the importance of considering GISTs in the differential diagnosis of obscure GI bleeding. A multidisciplinary approach is essential to ensure the best outcomes for these patients. Additionally, the use of minimally invasive surgery should be considered whenever possible to minimize postoperative complications and promote faster recovery.

## Introduction

Gastrointestinal stromal tumors (GISTs) are the most common type of soft tissue sarcoma found in the gastrointestinal (GI) tract. These tumors arise from the interstitial cells of Cajal (ICC), which are responsible for regulating GI motility [1]. GISTs can occur at any age but peak in the sixth decade of life [2]. The estimated annual occurrence of GISTs in the United States is 0.68 to 0.78 per 100,000 individuals, with the stomach being the most common site, accounting for 60% of cases, and the small intestine being the second (20%-30%) [3,4].

The majority of GISTs are caused by activating mutations in KIT or PDGFRA genes, which encode the receptor tyrosine kinases that regulate the ICCs [5]. GISTs of the small intestine pose a diagnostic challenge due to their location and may present with obscure GI bleeding and anemia, requiring a thorough investigation to detect the source. GISTs may present with vague and nonspecific symptoms, and can also be asymptomatic in some cases [4].

Early diagnosis and treatment of GISTs are essential for a good prognosis, as they can be aggressive and have the potential to metastasize to other organs. Surgical resection is the mainstay of treatment for localized GISTs, while imatinib mesylate, a tyrosine kinase inhibitor, is used for adjuvant therapy and for metastatic or unresectable diseases with significantly improved survival rates [3,4]. In addition to imaging studies, endoscopic techniques such as capsule endoscopy and balloon-assisted enteroscopy have been used. However, due to their rarity and nonspecific symptoms, diagnosis is often delayed, and GISTs are frequently found incidentally [6].

We report the case of a 74-year-old male who presented with anemia and obscure GI bleeding. The initial evaluation did not reveal the source; endoscopic and angiographic studies were inconclusive. It was not until a capsule endoscopy and then balloon-assisted enteroscopy was performed that a GIST in the jejunum was detected. The mass was resected via laparoscopic surgery, and a pathology report confirmed the diagnosis of a low-grade spindle cell GIST with free margins.

GISTs are typically treated with surgery, and the prognosis is largely dependent on the tumor size, mitotic rate, and tumor location [3]. Adjuvant therapy with tyrosine kinase inhibitors, such as imatinib, may be considered for high-risk GISTs or for cases with residual disease after surgery [6]. In this case, due to the low-risk nature of the tumor, adjuvant therapy was not recommended.

## Case presentation

A 74-year-old man with a medical history of albinism and type 2 diabetes, who was taking metformin 1850 mg and empagliflozin 125 mg, was admitted to the emergency department due to general malaise, nausea, vomiting, and bloody stools for the past 72 hours. Upon arrival, the patient had a stable blood pressure of 128/68 mmHg, heart rate of 94 bpm, respiratory rate of 17 rpm, temperature of 36.2°C, and blood oxygen saturation of 92%. He was alert and oriented, but pale. No respiratory symptoms were noted, and his abdomen was soft and non-tender. Digital rectal examination was positive for melena. Laboratory results showed leukocytosis (13360/mm3), severe anemia (hemoglobin of 6.9 g/dL and hematocrit of 20.7%), normal coagulation times, preserved renal function (creatinine 0.93 mg/dl, glomerular filtration rate (GFR): 86 ml/min/1.73 m²), and hyperlactatemia (4.48 mmol/L).

The patient was admitted to the intensive care unit, received a transfusion of red blood cell concentrates, and was started on intravenous omeprazole (40 mg) immediately and every eight hours. Upper endoscopy reported no lesions and colonoscopy showed abundant hematic debris; neither could identify the source of bleeding. A mesenteric angio-CT was performed which did not show active bleeding. We proceeded with a capsule endoscopy and bleeding was found at the level of the proximal jejunum. A balloon-assisted enteroscopy was then scheduled, finding an ulcerated subepithelial lesion with adherent clots suspected to be a GIST. The mass was tattooed and a diagnostic laparoscopy was planned. 

**Figure 1:**
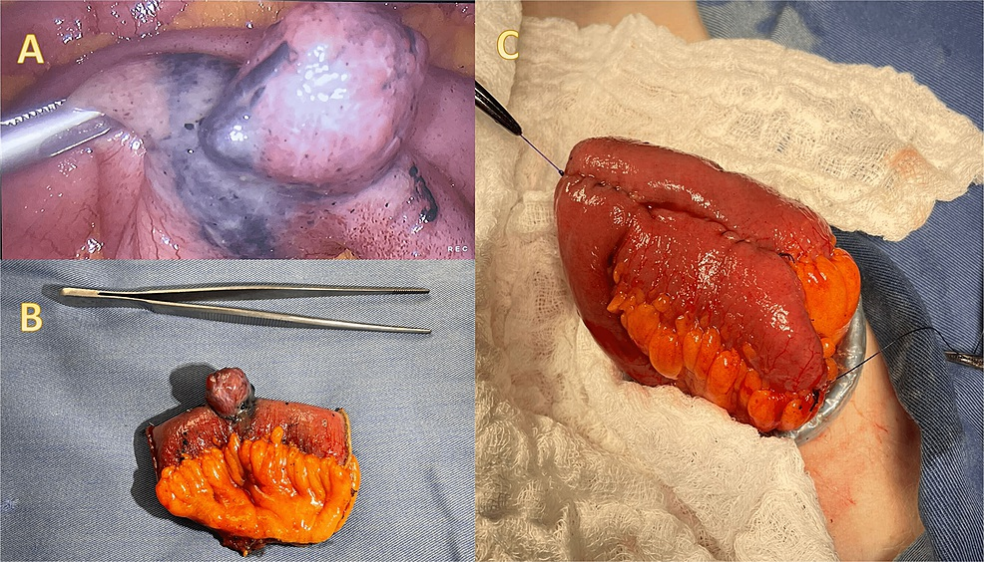
A. Laparoscopic view of the tumor marked in its base with an endoscopic tattoo. B. Surgical specimen. C. Laterolateral entero-entero anastomosis

Laparoscopy revealed a 2.5 cm well-demarcated indurated round mass 3 meters from the ileocecal valve. The segment containing the tumor was resected, and a latero-lateral anastomosis was performed as seen in Figure 1. Pathology report showed a 2 x 1.5 x 1.8 cm low-grade spindle cell GIST with free margins with a mitotic rate of ≤5 mitoses/50 high-power fields (HPF).

The patient was discharged on the fourth postoperative day with instructions to follow a soft diet and having demonstrated normal intestinal transit. There were no postoperative complications reported. The patient is currently being monitored according to institutional policies, and no tumoral activity or new episodes of bleeding have been reported.

## Discussion

GISTs are the most common type of soft tissue sarcoma (STS) found in the GI tract. They are primarily caused by activating mutations in KIT or PDGFRA genes. The stomach is the most common site for GISTs in the GI tract, accounting for 60% of cases, with the small intestine being the second (20%-30%) [3,4].

There is limited information available on the incidence of small bowel GISTs by region, as most studies report on the incidence of GISTs in general, without distinguishing by location. However, a few studies have reported the incidence of small bowel GISTs specifically. A study conducted in the United States found these accounted for approximately 10% of all GISTs, with the majority occurring in the ileum [6]. Another Japanese review reported a slightly higher incidence of approximately 15% with a slightly higher proportion in the jejunum [2]. A Spanish report has similar findings [5]. We could not find specific numbers for Latin America. 

Individuals suspected to have GISTs may experience various signs and symptoms, such as early satiety, postprandial pain, abdominal distension, bleeding to the abdominal cavity with acute abdomen, digestive tract bleeding, or exhaustion due to anemia [3,7]. Although risk factors for the development of GISTs have been studied, no environmental factors were identified. While there is a familial predisposition associated with germline mutations of KIT or PDGFRA, these are rare occurrences [4].

When a mass is identified and confirmed or suspected to be a GIST, surgical resection is the primary treatment option for patients with localized or potentially resectable disease, if it can be performed with minimal risk of complications. While local recurrence of GISTs is infrequent, avoiding rupturing or fracturing the tumor is crucial since violating the pseudocapsule is a significant factor in recurrence. While GISTs typically have a benign behavior, small bowel GISTs tend to be more aggressive with a three-year progression-free survival rate of 86.1% [3,8]. MItotic rate and tumor size are important prognostic factors for determining the risk of metastases and recurrence. In this case, tumor size was 2 cm, and mitotic rate was ≤5 mitoses/50 HPFs, giving a metastatic risk of 0% according to data from National Comprehensive Cancer Network (NCCN) guidelines, so the recommendation for imaging follow-up in this patient was to perform an abdominopelvic CT every 3-6 months for 3 to 5 years, then once a year [3]. 

A quarter of GISTs present with GI bleeding and anemia due to continuous blood loss. High suspicion is necessary, especially in those patients in whom upper and lower endoscopy are negative, as in this case [9]. This subgroup of patients with obscure GI bleed presents a diagnostic challenge that requires the use of additional methods that include capsule endoscopy, which some report as the first-line procedure after negative endoscopy. The next step would be deep endoscopy techniques that include balloon-assisted enteroscopy. Depending on the resources available, imaging studies can be employed, such as CT angiography, intravenous contrast-enhanced multidetector row CT, and magnetic resonance enterography [1].

CT is a sensitive imaging modality for detecting small GISTs, with a reported accuracy of up to 95% for lesions larger than 2 cm in diameter [10]. However, the sensitivity for detecting smaller ones decreases to approximately 50% [11]. In this patient, imaging studies could not identify the lesion; even in the retrospective revision of the CT after surgical resection, the mass was not spotted. 

## Conclusions

In conclusion, small bowel GISTs are a rare but important cause of GI bleeding and anemia. Diagnosis can be challenging, and a high degree of suspicion is required in patients with unexplained GI bleeding. Capsule endoscopy and balloon-assisted enteroscopy are valuable diagnostic tools for identifying the site of obscure GI bleeding. Surgical resection remains the mainstay of treatment for small bowel GISTs, with the goal of achieving complete resection with negative margins. The use of a multimodal approach, as in this case, aids in achieving disease control by using minimally invasive techniques and thus improving patient outcomes.
